# The State-of-the-Art Antibacterial Activities of Glycyrrhizin: A Comprehensive Review

**DOI:** 10.3390/microorganisms12061155

**Published:** 2024-06-06

**Authors:** Ru-Yi Chen, Jin-Jin Shi, Yan-Jun Liu, Jing Yu, Chang-Yun Li, Fan Tao, Jia-Feng Cao, Guan-Jun Yang, Jiong Chen

**Affiliations:** State Key Laboratory for Managing Biotic and Chemical Threats to the Quality and Safety of Agro-Products, School of Marine Sciences, Ningbo University, Ningbo 315211, China2311130039@nbu.edu.com (J.-J.S.); 2201130072@nbu.edu.cn (Y.-J.L.); 20102060117@lsu.edu.cn (J.Y.); 2211130023@nbu.edu.cn (C.-Y.L.); 2111091161@nbu.edu.cn (F.T.); caojiafeng@nbu.edu.cn (J.-F.C.)

**Keywords:** glycyrrhizin, antibacterial effect, licorice, HMGB1

## Abstract

Licorice (*Glycyrrhiza glabra*) is a plant of the genus Glycyrrhiza in the family *Fabaceae*/*Leguminosae* and is a renowned natural herb with a long history of medicinal use dating back to ancient times. Glycyrrhizin (GLY), the main active component of licorice, serves as a widely utilized therapeutic agent in clinical practice. GLY exhibits diverse medicinal properties, including anti-inflammatory, antibacterial, antiviral, antitumor, immunomodulatory, intestinal environment maintenance, and liver protection effects. However, current research primarily emphasizes GLY’s antiviral activity, while providing limited insight into its antibacterial properties. GLY demonstrates a broad spectrum of antibacterial activity via inhibiting the growth of bacteria by targeting bacterial enzymes, impacting cell membrane formation, and altering membrane permeability. Moreover, GLY can also bolster host immunity by activating pertinent immune pathways, thereby enhancing pathogen clearance. This paper reviews GLY’s inhibitory mechanisms against various pathogenic bacteria-induced pathological changes, its role as a high-mobility group box 1 inhibitor in immune regulation, and its efficacy in combating diseases caused by pathogenic bacteria. Furthermore, combining GLY with other antibiotics reduces the minimum inhibitory concentration, potentially aiding in the clinical development of combination therapies against drug-resistant bacteria. Sources of information were searched using PubMed, Web of Science, Science Direct, and GreenMedical for the keywords “licorice”, “Glycyrrhizin”, “antibacterial”, “anti-inflammatory”, “HMGB1”, and combinations thereof, mainly from articles published from 1979 to 2024, with no language restrictions. Screening was carried out by one author and supplemented by others. Papers with experimental flaws in their experimental design and papers that did not meet expectations (antifungal papers, etc.) were excluded.

## 1. Introduction

Infections have been a major cause of disease throughout human history, and bacterial infections pose a serious threat to human health, which can lead to significant morbidity and mortality [1]. Bacterial infections can cause inflammation in a variety of organs, such as glomerulonephritis caused by streptococcal and staphylococcal infections, a large proportion of which develop into chronic kidney disease or end-stage renal disease [2,3,4]. *Streptococcus pneumoniae* and *Staphylococcus aureus* are common in the incidence of influenza–bacterial co-infections in humans, and bacterial co-infections can lead to disease-worsening events that can be severe and even lead to death [5]. With increased susceptibility to bacterial infections, multiple myeloma occurs more frequently. Pneumonia due to *Klebsiella pneumoniae*, urinary tract infections due to Gram-negative bacterial infections, and acute bacterial infections may evolve into multiple myeloma [6]. Strongly effective drugs for the treatment of bacterial infections are needed due to several drawbacks of antibiotics active against multidrug-resistant bacteria [7]. Not to be overlooked, antibiotics still play an important role in organ transplantation and other surgeries as well as in diseases caused by bacteria. However, with the emergence of drug resistance, the inhibitory effect of some pathogenic bacteria on antibiotics gradually diminishes or even disappears. Therefore, it is necessary to discover new therapeutic agents that can effectively inhibit bacterial diseases [8]. Meanwhile, the research and development of broad-spectrum drugs is also underway, and the control of bacterial diseases will not be limited to antibiotics in the future. Screening programs of herbal medicines used for different diseases are in order, and people are always looking for other safe, green, and sustainable medicines to replace the use of antibiotics.

Herbal medicine has a long history in China, and in vitro antimicrobial experiments with herbs have been ongoing since 1950 [9]. Currently, most herbal medicines are mainly obtained from plants with a wide variety of species, and herbal medicines do not cause negative impacts such as environmental pollution. The active ingredients contained in herbal medicines are primarily the compounds originating from secondary metabolism (although some products of primary metabolism can also be considered as main active principles). These may include alkaloid, phenolic, and terpenoid compounds [10,11], which can improve the immune function, antibacterial, and antiparasitic abilities of the human body. In addition, herbal medicines do not develop resistance to pathogenic bacteria. At present, according to the social requirements for green and healthy development, the search for low-pollution and low-cost medicines has become one of the hotspots, and Chinese herbal medicines can fulfill these conditions. Some Chinese medicines and their active ingredients have shown strong immunomodulatory activities in higher organisms and their safety has been widely verified [12]. Feng X’s study found that Chinese herbs had a better inhibitory effect on bacteria such as *Streptococcus*, *Staphylococcus*, and *Pseudomonas* [13]. At present, based on the social requirements for green health development, it is urgent to seek low-pollution and low-cost drugs that can inhibit bacterial diseases. 

Licorice root is derived from *Glycyrrhiza glabra* L. It also can be extracted from *Glycyrrhiza uralensis* Fisch and *Glycyrrhiza inflata* Bat [14,15]. *Glycyrrhiza* glabra is widely distributed around the world, is of consistent quality, and is an excellent cultivar. It contains many naturally occurring active compounds as well as and GLY, an oleanane-type pentacyclic triterpenoid saponin compound isolated from licorice, which can also be used as a sweetener [16,17,18]. GLY is metabolized by glucuronidase in the gastrointestinal tract to GLY after oral administration to humans and rats and can be completely absorbed [19]. Meanwhile, GLY has a wide range of pharmacological and biological activities [20], including anti-inflammatory, immunomodulatory, hepatoprotective and neuroprotective, antitumor, anti-arthritic, antiviral, and anticancer properties [21,22]. GLY can modulate certain enzymes related to oxidative stress and inflammation, and can downregulate certain pro-inflammatory cytokines, which protects against reactive oxygen species (ROS) or inflammation-induced injury. In this paper, we focus on the mechanism of action of bacterial diseases on the human body and propose an effective inhibitory mechanism of GLY.

## 2. Pharmacological Activity of GLY 

Since the 1950s, scholarly articles on GLY have been systematically published, with a notable exponential increase in volume observed throughout the 21st century. In recent years, there has been a discernible surge in research endeavors investigating GLY. Clinical reports indicate its utilization in the treatment of various conditions including hepatitis, bronchitis, gastric ulcers, AIDS, certain cancers, and dermatological disorders [23]. GLY has been used for treating hepatitis for more than 20 years in Japan [24]. Miyake et al. found that a daily injection of GLY can reduce alanine aminotransferase (ALT) levels in patients with chronic viral hepatitis in a dose-dependent manner, and the intravenous injection of GLY can significantly reduce serum liver enzymes and improve liver tissue [25]. Zhang et al. also verified that a daily intravenous injection containing a certain dose of GLY is a safe and effective treatment method for reducing or normalizing ALT levels in patients with chronic hepatitis B [18,25]. Sun et al. demonstrated that GLY improves liver dysfunction in patients with chronic hepatitis B by affecting the extracellular secretion of the hepatitis B surface antigen, ultimately improving the immune status of hepatitis B virus (HBV) patients and having a direct effect on combating HBV [18]. In addition, in patients with chronic hepatitis B, Eisenburg et al.’s study found that the intravenous injection of GLY for one year can have a positive impact on the development of the disease, with a success rate of 30–40%, which is comparable to the results obtained with interferon [26]. Hence, there is a prevalent consensus that pharmaceutical injections containing GLY preparations exhibit efficacy in attenuating liver necrosis and inflammation among patients with chronic hepatitis C. Given GLY’s natural origin, the sustained administration of these medications may offer potential prophylactic benefits against certain liver tissue diseases, including cirrhosis.

Concurrently, GLY exhibits promising therapeutic efficacy in various conditions, including type 2 diabetes mellitus (T2DM). T2DM, a leading cause of global mortality, is characterized by hyperglycemia, chronic insulin resistance, and β-cell dysfunction, constituting a chronic metabolic disorder with progressive deterioration in cellular function and insulin secretion [27]. While T2DM remains incurable, its management focuses on glycemic control and symptom alleviation, albeit often accompanied by noticeable drug-related side effects. Hence, exploring alternative pharmacotherapeutic avenues assumes paramount importance. Tan et al.’s research elucidated GLY’s multifaceted benefits in T2DM management, encompassing reductions in blood sugar and insulin levels, the amelioration of insulin resistance and glucose tolerance, the modulation of lipid metabolism, and the augmentation of insulin secretion. Furthermore, GLY demonstrates favorable outcomes in cardiovascular and cerebrovascular diseases [28]. Notably, its efficacy extends to conditions like allergic asthma, wherein GLY evinces minimal adverse reactions. Fouladi et al. demonstrated that GLY significantly inhibits OVA-induced eosinophil proliferation in lung and airway tissues. In addition, GLY can also alleviate inflammation by regulating high-mobility group box 1 (HMGB1) [29]. Wu et al. investigated the therapeutic effect of GLY on spinal cord injury (SCI) and found that GLY inhibits HMGB1 through the p38 mitogen-activated protein kinase (p38 MAPK)/c-Jun N-terminal kinase (JNK) signaling pathway, thereby reducing the inflammatory response after SCI, thus possessing therapeutic potential for SCI [30]. 

GLY boasts an extensive research legacy in the realm of liver diseases, enjoying widespread utilization in their treatment owing to the minimal adverse effects and pronounced therapeutic efficacy of herbal remedies. Notably, at elevated concentrations that remain non-toxic to cells, GLY demonstrates inhibitory properties against the replication of various diseases, including Japanese encephalitis, mammalian tick-borne encephalitis, and yellow fever [31]. 

## 3. In Vitro Antimicrobial Effect of GLY

Since the glory days of antibiotics in the mid-20th century, natural products have become effective therapies against pathogenic bacteria [32,33]. Herbal medicines are renowned for their ability to inhibit bacterial growth or pathogenicity and regulate specific genes associated with extracellular virulence. Presently, chemical interventions such as antibiotics remain the primary approach to bacterial infections. However, the misuse and overuse of antibiotics have precipitated the emergence of drug-resistant strains, alongside environmental and human health concerns such as antibiotic residues [34]. Herbal medicines, possessing inhibitory and bactericidal effects, fortify the body’s defenses without fostering drug resistance. GLY, the principal active compound in licorice, showcases a diverse array of therapeutic properties including anti-inflammatory, antitumor, antioxidant, antiviral, antibacterial, hepatoprotective, and immunomodulatory effects. The reliance on antibiotics has exacerbated the critical issue of bacterial drug resistance, prompting a shift towards the utilization of Chinese herbs in disease management. Nonetheless, the antibacterial efficacy of Chinese herbs still lags behind that of antibiotics. 

### 3.1. GLY Achieves Antibacterial Effects by Inhibiting Enzyme Activity

**Figure 1:**
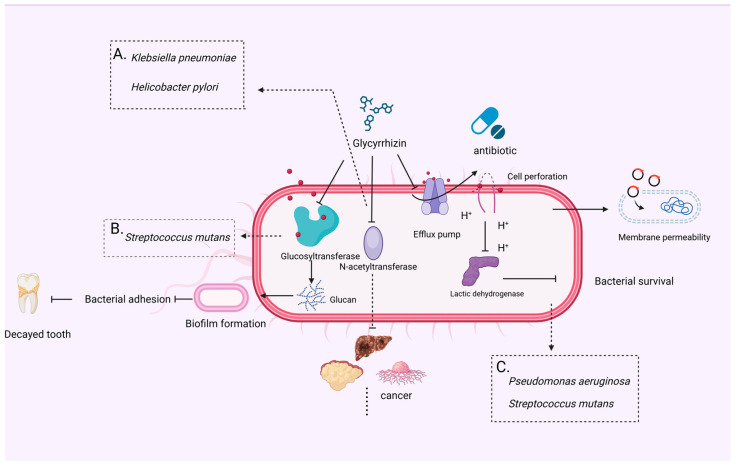
In vitro antimicrobial mechanism actions of GLY. (**A**) Effect of GLY on NAT activity in bacteria in vivo, (**B**) effect of GLY on biofilm formation, (**C**) effect of GLY on bacterial cell membrane permeability. This figure was produced by Biorender (Agreement number: NN26VOKDMK).

*Klebsiella pneumoniae* represents a prevalent etiological agent of antimicrobial-resistant opportunistic infections in hospitalized individuals [35]. The inherent resistance of this species to penicillin, coupled with the widespread acquisition of resistance to multiple antimicrobial agents within its population, underscores the challenges posed by this pathogen in clinical settings [36]. *Helicobacter pylori* is a major cause of peptic ulcer, gastritis, and gastric cancer [37,38]. The resistance of *H. pylori* to clarithromycin is the main reason for the failure of drug therapy. GLY demonstrates significant inhibitory effects against both *K. pneumoniae* [39] and *H. pylori* [40], suggesting its potential therapeutic role in combating peptic ulcers and other gastric diseases. GLY inhibits the activity of aromatic amine N-acetyltransferase (NAT), both in cytoplasmic preparations and in whole-cell assays, with dose-dependent effects noted in studies using high-performance liquid chromatography (HPLC) (Figure 1A). In the case of *H. pylori*, GLY’s inhibition of NAT may play a key role in its ability to reduce bacterial resistance to clarithromycin, a common issue in conventional treatment [40]. GLY has demonstrated antiviral, immunomodulatory, and anti-inflammatory properties, positioning it as a potential prophylactic agent for gastric diseases [37,41,42,43]. It rapidly eliminates both clarithromycin-resistant and susceptible isolates of *H. pylori* [44]. This bactericidal activity may be linked to the inhibition of aromatic amine NAT, an enzyme in *H. pylori* involved in the metabolic activation of chemical carcinogens [40]. Krahenbuhl et al. found that orally administered GLY is almost completely hydrolyzed by intestinal bacteria and reaches systemic circulation in the form of GLY [45,46]. Chung et al. determined NAT activity by acetyl coenzyme, a cycling assay, and HPLC, and revealed that GLY had a dose-dependent bactericidal effect in *H. pylori* cultures [40]. Co-treatment of GLY with *H. pylori* revealed that the apparent values of K_m_ and V_max_ were reduced, indicating that GLY inhibits NAT activity in *H. pylori* (Figure 1A).

### 3.2. GLY Affects the Formation of Bacterial Cell Membrane

GLY, characterized as an organic weak acid, targets the cell membrane of *Streptococcus mutans*, leading to alterations in membrane permeability. This results in an influx of protons into the cell, subsequently reducing intracellular acidity. Additionally, GLY inhibits glycolysis-related enzymes, effectively impeding the glycolytic process and thereby restricting the growth of *S. mutans* [47]. Liu et al. found that GLY exhibits a significant and dose-dependent inhibition of both the proliferation and acid production of *S. mutans* [48]. Dental caries, characterized by the progressive destruction and loss of tooth enamel, is primarily caused by *S. mutans*, making it a significant oral health concern. GLY exhibits an inhibitory effect on *S. mutans*, thereby contributing to the protection of the gingival environment [49]. Segal et al.’s study demonstrated that high concentrations of GLY slightly impedes bacterial growth, while low concentrations of GLY prevents *S. mutans* from adhering to teeth and forming plaques, thus reducing caries formation. This inhibition is attributed to GLY’s ability to inhibit glucosyltransferase (GTase), an enzyme crucial for glucan production, which is essential for bacterial adhesion [50]. Similarly, Ham et al.’s research corroborated the inhibitory impact of GLY on *S. mutans*. Through screening various plant extracts, they identified GLY as a compound that, when combined with other extracts, inhibits *S. mutans* cell growth, biofilm formation, and glucan synthesis by GTase. Consequently, this inhibitory action impedes dental biofilm formation, as illustrated in their findings [47] (Figure 1B). 

### 3.3. GLY Affects the Permeability of Bacterial Cell Membranes

Chakotiya et al.’s study highlighted the effective inhibitory effect of GLY on *Pseudomonas aeruginosa*. This bacterium is implicated in microbial keratitis, a serious eye infection that poses a significant threat to vision. *P. aeruginosa* infection is a major cause of keratitis and is associated with various risk factors. While topical antibiotics are commonly used to manage *P. aeruginosa* keratitis by reducing bacterial load, the emergence of resistant strains presents a formidable challenge to treatment efficacy [51]. Hazlett et al. further elucidated the mechanisms underlying *P. aeruginosa* resistance. They found that resistance in *P. aeruginosa* primarily arises from the overexpression of bacterial membrane permeability, efflux pumps, and antibiotic-inactivating enzymes. These mechanisms impede the efficacy of antibiotic treatment [52]. *P. aeruginosa* presents additional challenges due to its low permeability of the cell wall and cell membrane. Consequently, antibiotics face difficulty in penetrating these barriers to reach their target sites. Moreover, the activity of efflux pumps further reduces the effectiveness of antimicrobial agents by removing them from the bacterial cell, thereby diminishing their toxic effects. These factors collectively contribute to the difficulty in treating *P. aeruginosa* infections. Chakotiya et al. conducted a study examining various licorice extracts containing compounds such as alkaloids, flavonoids, tannins, saponins, and coumarins, as well as purified GLY, for their antibacterial properties [51]. They found that both the extract and GLY had strong anti-Pseudomonas activity. The study also employed flow cytometry, coupled with a microdroplet biofilm formation test, to assess cell membrane formation and permeability. It was observed that GLY induces alterations in bacterial membrane permeability, leading to a reduction in bacterial viability. Furthermore, GLY is found to inhibit efflux pump activity, which contributes to a reduction in drug resistance in bacteria. These results suggest that GLY’s mechanism of action involves modulating bacterial membrane permeability and efflux pump activity, thereby enhancing its antibacterial efficacy against *Pseudomonas* species (Figure 1C). 

### 3.4. GLY Sensitizes the Antibacterial Activities of Other Antibacterial Agents 

**Figure 2:**
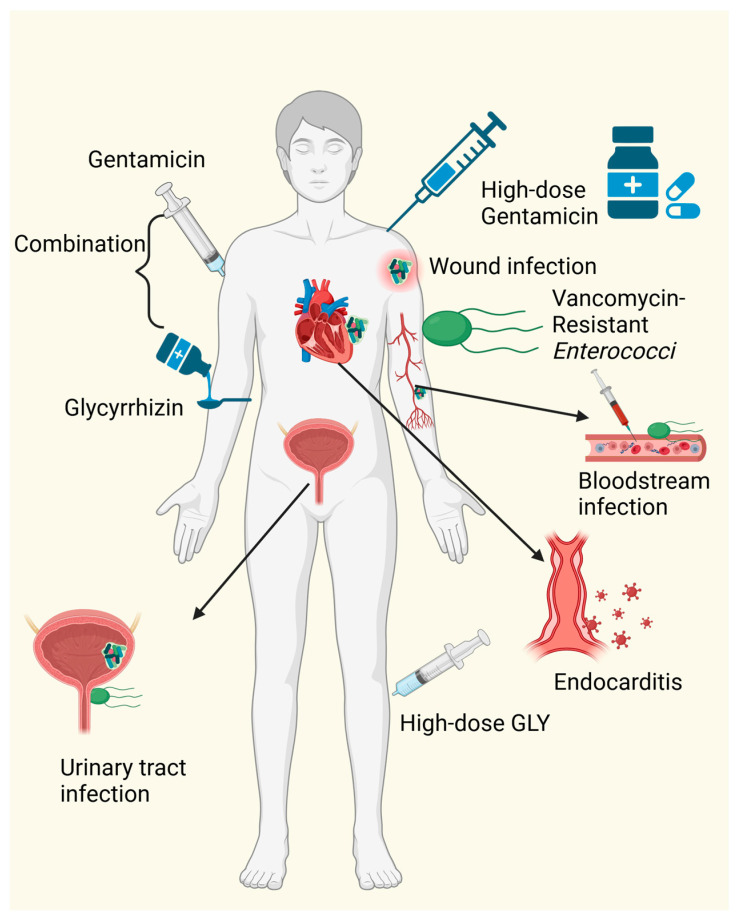
The synergistic antibacterial mechanisms of GLY. This figure was produced by Biorender (Agreement number: CP26WJH8RI)).

GLY possesses a structural advantage in self-conjugating to form a cyclic conformation, creating a spherical inner space capable of encapsulating other medicinal compounds to form inclusion complexes. This property serves as a drug delivery system, enhancing drug absorption [53]. GLY inclusion complexes can be utilized by a variety of compounds such as cholesterol [17], lagochiline [54], and paclitaxel [55,56]. The formation of inclusion complexes prolongs the action of drug compounds, enhances their stability, and reduces the therapeutic load/dose. The concurrent administration of this compound and antibiotics shows promise in reducing antibiotic resistance and effectively improving antibiotic efficacy. In a study by Schmidt et al., GLY, in combination with gentamicin, exhibits potential therapeutic effects against *Enterococci* (Table 1). At a sub-inhibitory concentration of 2.4 mM GLY, the MIC of gentamicin against vancomycin-resistant *Enterococci* decreases from 8–32 mg/L to 0.125–2 mg/L, demonstrating the therapeutic potential of GLY and gentamicin combination therapy against *Enterococci* (Figure 2).

## 4. In Vivo Antimicrobial Mechanism of GLY 

### 4.1. GLY Affects the Regulation of Immune Cells

#### 4.1.1. Macrophages

**Figure 3:**
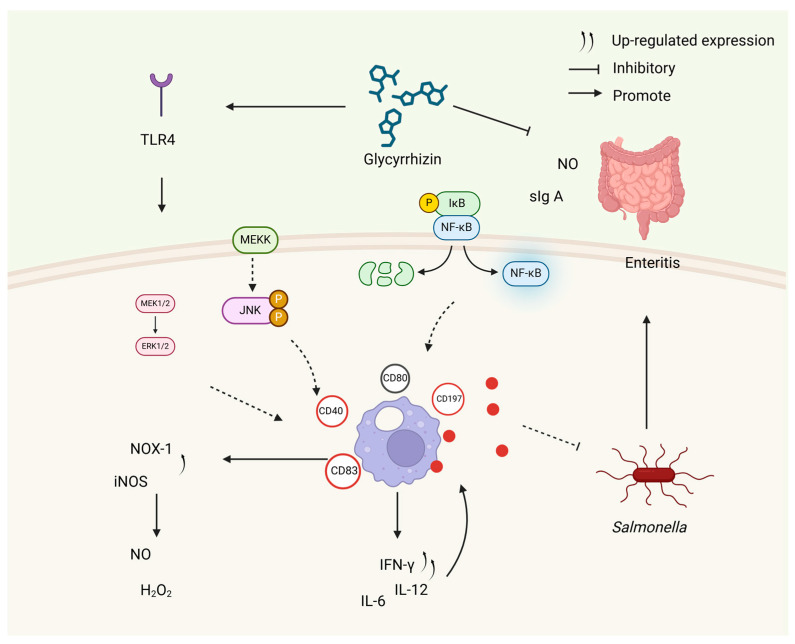
The effect of GLY on macrophage regulation. This figure was produced by Biorender (Agreement number: UM26WJFIFZ).

Managing *Salmonella enterica* in the intestinal tract presents a persistent challenge given its status as a significant contributor to foodborne illnesses. Wang et al. demonstrated that GLY increases the internalization of fluorescein isothiocyanate (FITC)–dextran and *S. enterica* in chicken macrophages and induces the expression of inducible nitric oxide synthase (iNOS) and NADPH oxidase-1 (NOX-1), which promotes the cellular production of nitric oxide and hydrogen peroxide [60]. Nitric oxide (NO) plays a pivotal role in combating invading pathogens, particularly intracellular ones, by exerting a potent bactericidal effect alongside reactive oxygen species (ROS). Studies have confirmed that nuclear factor-κB (NF-κB) and the JNK pathway regulate the gene expression responsible for NO production and interferon-gamma (IFN-γ) synthesis induced by GLY. These findings underscore GLY’s robust immunomodulatory properties in activating chicken macrophages and bolstering chickens’ ability to combat *S. enterica.* Paudel et al. proposed that GLY exhibits anti-inflammatory activity by reducing the mRNA expression levels of HMGB1, Toll-like receptor 4 (TLR4), NF-κB, and tumor necrosis factor alpha (TNF-α) [61]. Similarly, Chang CH et al. suggested that GLY prevents the bacterial invasion of macrophages, reduces the secretion of pro-inflammatory cytokines (such as IFN-γ, TNF-α, interleukin-6 (IL-6)), activates macrophages, and enhances the secretion of anti-inflammatory cytokines (such as IL-10), thus inhibiting *S. enterica* infections. The GLY-induced maturation of mouse bone marrow dendritic cells (BMDCs) is found to involve the NF-κB, extracellular signaling-associated protein kinases (ERK1/2), and p38 MAPK pathways. Furthermore, Chang CH et al. observed that GLY treatment significantly increases the expression of TLR2 (both mRNA and protein) while downregulating the gene expression of TLR3 and TLR4 in BMDCs, promoting signaling activation at the TLR4 receptor. The major downstream signaling pathways in the TLR-signaling-induced maturation of BMDCs were found to be MAPKs and NF-κB. Through inhibitor experiments, Chang CH et al. demonstrated that GLY induces the maturation of BMDCs via the NF-κB, ERK, and p38 MAPK signaling pathways, leading to the increased production of NO and H_2_O_2_ and the expression of IFN-γ (Figure 3).

#### 4.1.2. Neutrophils

**Figure 4:**
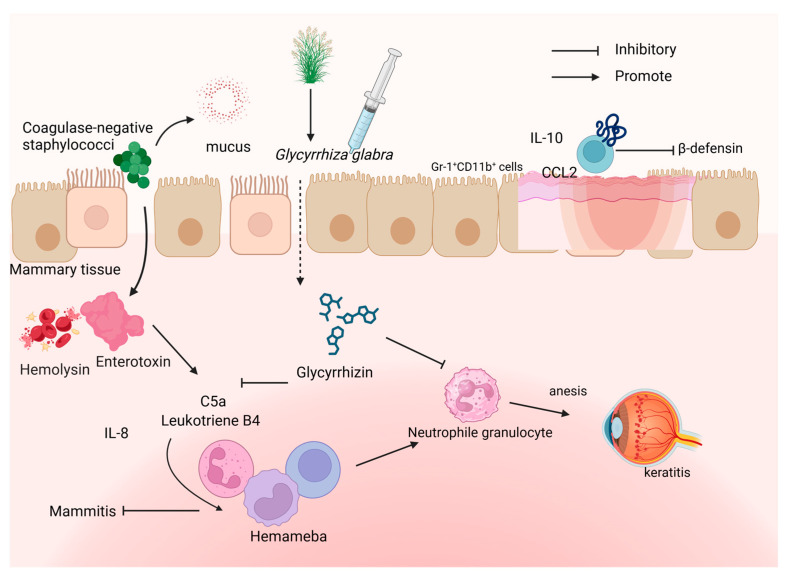
The effect of GLY on neutrophil regulation. This figure was produced by Biorender (Agreement number: IC26WJGXPC).

Pathogenic bacteria invade the mammary gland, attach to the mammary tissue, and then grow to produce bacterial toxins [62]. *Coagulase-negative staphylococci* were isolated from milk during intramammary infection [62,63]. Following treatment with GLY, it is observed that the intramammary infusion of GLY decreases the migration of leukocytes, particularly neutrophils, to the affected mammary glands. This treatment also induces IL-12 production and enhances CD4^+^ T-helper-1-type cytokines. These effects were confirmed by monitoring bacterial counts and somatic cell counts (SCCs), as well as alpha-lactalbumin, lactoferrin, and histamine concentrations in the milk. These changes facilitated a cell-mediated immune response (Figure 4). 

A study of a mouse model of burn wound infection was used to investigate how *P. aeruginosa* affects burned tissue [64]. The burned mice exhibited decreased resistance, leading to *P. aeruginosa* infection at the wound site. Murine β-defensin production was identified as a key factor in resisting such infections [65]. Yoshida et al. found that reduced murine β-defensin levels are associated with a higher risk of burn wound infection. Gr-1^+^CD11b^+^ cells at the edges of burned tissue were shown to inhibit antimicrobial peptide production by releasing inhibitory factors like CCL2 and IL-10, which hinder peptide production and facilitate bacterial invasion and growth [66,67] (Figure 4). 

### 4.2. GLY Mediates Immune Regulation of Non-Immune Cells

#### 4.2.1. GLY Induces Autophagy

**Figure 5:**
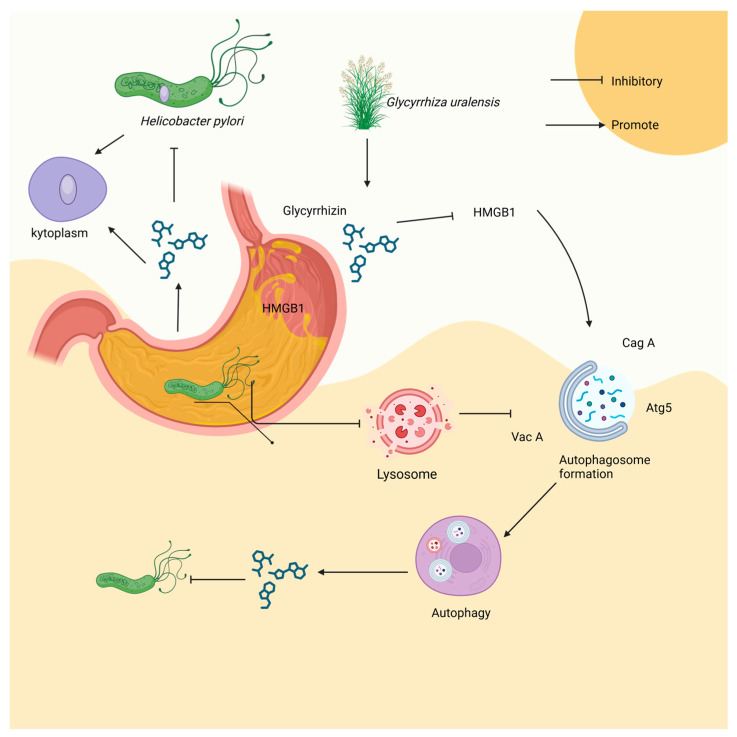
The effect of GLY on autophagy. This figure was produced by Biorender (Agreement number: MR26WJE453).

GLY has been documented to attenuate lung injury (ALI) by inducing cellular autophagy and increasing the number of autophagosomes in an LPS-induced ALI mouse model [68]. A recent study also found that GLY could suppress *H. pylori* infection [69]. To be specific, *H. pylori* infection leads to the secretion of effector proteins such as CagA and VacA, which inhibit autophagy via disrupting lysosomal permeability, impairing the autophagy–lysosomal pathway, resulting in an increase in HMGB1 protein levels and promoting the survival of the bacteria within the host. GLY induces autophagy by inhibiting HMGB1, thereby restoring lysosomal membrane integrity, promoting autolysosome formation, and inhibiting HMGB1-induced lysosomal membrane permeabilization (LMP). This restoration of autophagy and lysosomal degradation is accompanied by a reduction in inflammation (Figure 4). Overall, these studies highlight the potential therapeutic role of GLY in mitigating the effects of *H. pylori* infection and ALI through its modulation of autophagy and inflammation pathways (Figure 5).

#### 4.2.2. GLY Inhibits Pyroptosis

**Figure 6:**
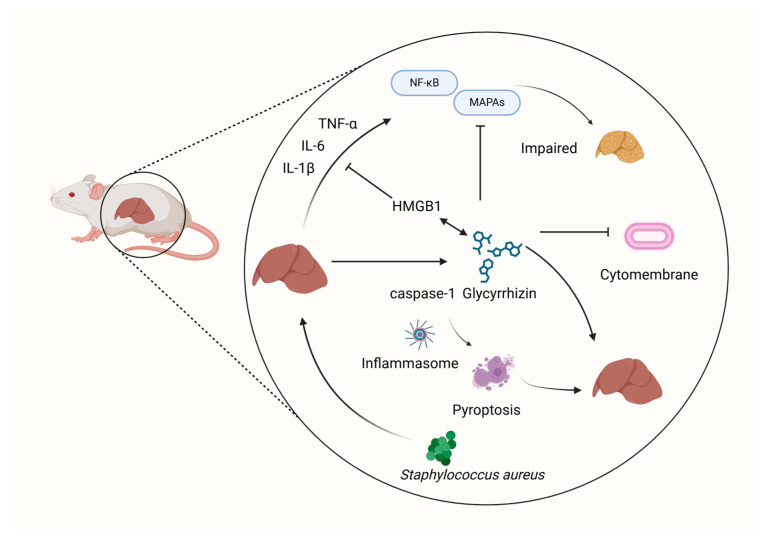
The effect of GLY on pyroptosis. This figure was produced by Biorender (Agreement number: NM26WJG6WI).

*S. aureus*-induced ALI poses a significant clinical mortality risk. *S. aureus* triggers inflammatory vesicles, enhances caspase-1-induced pyroptosis, and releases mature IL-1β to promote inflammatory responses, ultimately leading to ALI. GLY is found to exert its protective effects by inhibiting the activation of the NF-κB and MAPK (Erk1/2 and p38) signaling pathways [70]. Additionally, GLY is observed to directly bind to HMGB1, inhibiting its activity, as confirmed through inhibitor assays and to reduce serum and blood levels in *S. aureus*-infected mice serum and lung tissue production of pro-inflammatory cytokines (IL-6, TNF-α, IL-8, IL-1β) [71] (Figure 6). 

#### 4.2.3. GLY Inhibits NF-κB and MAPK Signaling Pathways in Which HMGB1 Is Involved

The NF-κB and MAPK signaling pathways are primarily involved in the inflammatory response. NF-κB, a major intracellular transcription factor, typically exists as an inactive cytoplasmic complex bound to its inhibitor protein IκB. Upon IκB degradation, NF-κB translocates to the nucleus, facilitating the expression of pro-inflammatory cytokines [72]. The MAPK signaling pathway comprises JNK/stress-activated protein kinases (SAPKs), p38, and extracellular-signaling-associated protein kinases (ERK1/2) [73]. Numerous inflammatory stimuli activate both NF-κB and MAPK pathways, leading to the increased production of pro-inflammatory cytokines and chemokines [74]. HMGB1, an alert protein, amplified inflammation in *P. aeruginosa*-induced keratitis [58,75]. A previous study revealed that GLY is a natural inhibitor of HMGB1 [76,77]. GLY’s mechanism of action involves inhibiting the release of HMGB1, thereby suppressing the activation of apoptosis-associated proteins and reducing the expression of pro-inflammatory factors. Research suggests that GLY achieves this by binding to HMGB1, which subsequently leads to a reduction in the expression of inflammatory factors. This reduction is primarily attributed to the inhibition of HMGB1 phosphorylation, which hinders its transport and release. Additionally, GLY blocks the interaction between HMGB1 and receptor of advanced glycation endproducts (RAGE), consequently inhibiting the expression of TLR4 [78]. In keratitis models, HMGB1 has multiple functions involved in autocrine and paracrine positive feedback mechanisms. HMGB1 interacts with Toll-like receptors (TLRs) and RAGE, triggering a downstream inflammatory cascade that activates NF-κB, thereby up-regulating the expression of pro-inflammatory mediators. The utilization of GLY in keratitis treatment has shown promising results. GLY binds to HMGB1, leading to a reduction in corneal disease progression. By primarily reducing HMGB1 levels, GLY helps diminish the expression of pro-inflammatory factors such as IL-1β and C-X-C motif chemokine ligand 2 (CXCL2) (both mRNA and protein). Moreover, GLY treatment enhances the levels of antimicrobial proteins like CRAMP and Methyl-CpG binding domain protein 2 (MBD2), contributing to a protective effect against *P. aeruginosa*-induced keratitis. Peng et al. further verified that GLY improved keratitis prognosis through reducing the levels of experimental mRNA expression of *IL-1β*, *TNF-α*, *CXCL2*, and HMGB1 in vivo [57] (Figure 4). 

### 4.3. GLY Reduces Bacterial Infection through Intestinal Flora

The intestinal flora is crucial for combating *S. enterica* infection and supporting intestinal immunity. However, an *S. enterica* infection triggers intestinal inflammation and disrupts the microbial balance. A study found that *S. enterica* infection significantly increased the relative abundance of *Verrucomicrobia* and *Akkermansia* in the cecum [79] (Table 2), and similar results were obtained by Stecher et al. [80]. In a separate mouse study on chronic enteritis, Ganesh found that an increase in *Akkermansia*’s relative abundance is linked to heightened intestinal inflammation. Another study demonstrated that treating *S. enterica*-infected mice with GLY substantially lowers *Verrucomicrobia* levels [67]. GLY treatment is effective in mitigating *S. enterica* infection by restoring the intestinal flora’s balance and reducing *S. enterica*’s colonization in the ileum and colon, which, in turn, alleviates mucosal damage and decreases intestinal tract infection [67,81].

The antimicrobial mechanisms of GLY encompass anti-inflammation, immunomodulation, and antibacterial activity [83]. Studies investigating GLY’s antibacterial mechanisms against various pathogens such as *K. pneumoniae* [39], *S. aureus* [70], *H. pylori* [40], *S. enterica* [82], *Enterococcus* [59], and *P. aeruginosa* [51] have been conducted both in vitro and in vivo. 

The in vitro antimicrobial mechanism of GLY involves several key processes. First, GLY affects biofilm formation, which is crucial for bacterial colonization and resistance. Biofilms protect bacteria from antibiotics and the immune system, so disrupting their formation is a potent antimicrobial strategy. Second, GLY alters the permeability of bacterial cell membranes, impacting the integrity and function of these membranes, leading to bacterial cell death. Third, GLY inhibits GTase and aromatic NAT, enzymes involved in bacterial metabolism and survival. Moreover, studies have shown that the inhibitory effect of GLY on bacteria is dose-dependent, indicating that higher concentrations of GLY results in more substantial antibacterial effects. This characteristic is crucial in considering GLY for therapeutic use, especially for drug-resistant bacteria. Given these findings, GLY has a significant potential for use in combination with other antibiotics, as it may reduce the concentration of conventional drugs needed, enhancing their efficacy and potentially mitigating drug resistance. 

As previously noted, the in vivo antibacterial mechanism of GLY involves inhibiting the expression of HMGB1, reducing pro-inflammatory factors, and achieving anti-inflammatory effects. In the context of intestinal bacterial infections, GLY can also regulate the intestinal flora, aiding in the fight against bacterial invasion. Additionally, GLY resists bacterial infections through the formation of autophagosomes and the production of antimicrobial peptides, providing a multi-layered defense against bacterial pathogens. Overall, these in vitro and in vivo mechanisms suggest that GLY has a promising role in the development of new antimicrobial therapies, particularly when used in combination with other antibiotics to enhance efficacy and reduce the risk of drug resistance. 

## 5. Conclusions and Future Perspectives

Certain bacterial strains have developed significant resistance to antibiotics, impacting the treatment of infectious diseases. This underscores the urgent need for effective, affordable, and non-resistant medications. Chinese herbal medicine, renowned for its extensive historical usage, potent inhibitory effects, and lack of resistance, has emerged as a primary focus for research. There is a growing body of research on Chinese herbal medicine, supported by the fact that 80% of the global population relies on indigenous plant therapies [39]. However, it is important to acknowledge the potential for adverse reactions associated with herbal remedies. The utilization of licorice dates back to ancient civilizations such as the Greek and Roman empires. Both early Chinese and Western medical traditions have recognized licorice for its efficacy in addressing gastrointestinal issues, coughs, bronchitis, and arthritis. GLY, a naturally occurring compound found in licorice, holds significant medical promise and may contribute to the treatment and prevention of various ailments including viral infections and immune deficiencies. Its potential applications render GLY a subject worthy of further exploration and potential clinical use. Moreover, GLY can also enhance the efficacy of other drug molecules against pathogenic bacteria [39,51]. Several studies have shown that GLY significantly increases the solubility and affects the pharmacokinetic characteristics of supramolecular complexes via improving their permeability and stability and increasing their efficacy [84]. The amphiphilic nature of GLY allows it to form water-soluble couplings with a wide range of hydrophobic drug compounds, and such supramolecular complexes can increase the solubility of hydrophobic compounds by tens of times. It has been shown that increased drug solubility in the presence of GLY is accompanied by a significant decrease in the therapeutic dose of the drug [85]. The interaction of GLY with model lipid membranes was investigated by dynamic NMR and MD methods. It was found that GLY doped into lipid bilayers has an effect on the mobility of both the polar head and hydrophobic tail of lipids, and that GLY always carries some water molecules into the membranes, leading to an increase in the permeability of small-molecule membranes. Moreover, GLY is also used as a component in dressings used for bacteria-induced wounds. Li et al. demonstrated that GLY-based mixed hydrogel promotes the healing of uninfected skin wounds and the healing of skin wounds infected by *S. aureus* by enhancing the formation of granulation tissue, promoting collagen deposition, reducing bacterial infection, and downregulating the inflammatory response [86].

The frequent utilization of antimicrobial drugs fosters the emergence of multidrug-resistant microorganisms, posing significant threats to human health and food production. To address this challenge, the exploration of novel active ingredients and drug combinations is imperative [87]. Research shows that combining GLY with gentamicin results in synergistic effects against gentamicin-resistant Enterobacteriaceae [59]. Consequently, Schmidt et al. proposed further investigation into the in vivo combination of antibiotic compounds with saponins [59] and found that that employing extracts alongside antibiotic compounds can decrease the MIC of the respective antibiotics. These findings underscore the potential of combining GLY with existing antimicrobial agents to combat multidrug resistance effectively.

GLY, the main active ingredient in licorice, has been extensively studied for its applications in antimicrobial therapy [88]. Its in vitro antimicrobial mechanisms include the inhibition of bacterial enzymes, the influencing of the formation of bacterial cell membranes, alterations in permeability to inhibit bacterial growth, and the enhancement of the antimicrobial activities of other antibacterial agents. For example, GLY inhibits the activity of NAT in *H. pylori* and *K. pneumoniae* to exert its bactericidal effect. GTase synthesizes sticky dextran from sucrose and promotes the formation of oral biofilms and the attachment of oral microorganisms [47]. GLY is also demonstrated to inhibit the synthesis of cell membranes of *S. enterica* by abrogating GTase activity, thereby inhibiting the formation of dental organisms [25,48,49,89]. Even in the presence of sucrose, GLY markedly impedes adhesion (plaque formation) without impacting bacterial growth. Furthermore, GLY influences bacterial drug resistance by altering the permeability of the bacterial cell membrane, thus diminishing bacterial survival. Additionally, it diminishes drug resistance by reducing survival rates and efflux pump activity. GLY interacts with HMGB1 (AB box), impacting HMGB1/TLR4 signaling and stimulating signaling activation at the TLR4 receptor. This fosters the maturation of BMDCs, triggering downstream MAPK and NF-κB signaling pathways, resulting in increased NO and H_2_O_2_ production and IFN-γ expression. In *P. aeruginosa* keratitis, neutrophils play a pivotal role in phagocytosing and eliminating microorganisms. HMGB1 activates neutrophil-mediated inflammation and tissue damage via the TLR4 and RAGE pathways. During *P. aeruginosa* keratitis, activated keratocytes release chemoattractant cytokines like CXCL2 and CXCL5, recruiting neutrophils into the corneal stroma. Topical GLY treatment reduces CXCL2 expression, consequently lowering neutrophil infiltration. GLY also hampers the downstream pathway of Gr-1^+^CD11b^+^ cells in burned tissues by suppressing C-C motif ligand 2 (CCL2) and IL-10 production [63], while promoting β-defensin production against invading bacteria. Additionally, GLY improves lysosomal membrane permeability, enhances autolysosome formation, and mitigates intracellular *H. pylori* growth, leading to reduced inflammation [69].

HMGB1, identified in 1973 as a chromosomal non-histone protein, owes its nomenclature to its pronounced electrophoretic mobility in polyacrylamide gels. The dual HMG-box structural domains within HMGB1, referred to as the A box and the B box, constitute its DNA-binding regions. These domains, operating in tandem, have been recognized as potential sites for the binding of HMGB1 antagonists [90]. HMGB1 plays a multifaceted role in various cellular processes encompassing chromatin remodeling, nuclear transcription, replication, DNA repair, and nucleosome assembly [91]. Additionally, HMGB1 functions as a damage-associated molecular pattern molecule. It features two distinct nuclear localization sequences (NLSs), and the acetylation of multiple lysine residues within these NLSs facilitates the translocation of HMGB1 from the nucleus to the cytoplasm. Subsequently, HMGB1 is released into the extracellular milieu, where it interacts with receptors implicated in inflammation, cellular differentiation, cell migration, angiogenesis, tumor metastasis, and drug resistance [90]. When released extracellularly, HMGB1 exerts pro-inflammatory effects by stimulating various cells to express inflammatory mediators such as TNF-α and IL-1β, thereby initiating inflammatory responses and activating the NF-κB pathway. These pro-inflammatory actions have been implicated in conditions including keratitis [92], acute lung injury [93], arthritis [19], neuroinflammation [94], and meningitis [70], underscoring the pressing need for HMGB1 inhibitors. GLY, an HMGB1 inhibitor [95], is a prominent bioactive compound derived from the traditional Chinese herb *G. glabra*. GLY has demonstrated anti-inflammatory properties and has received FDA approval for the treatment of hepatitis, highlighting its therapeutic potential in attenuating HMGB1-mediated inflammation [93]. Epithelial–mesenchymal transition (EMT) is a biological mechanism wherein epithelial cells transition into mesenchymal phenotypes. HMGB1 has been implicated in inducing EMT and fostering cell proliferation and regeneration in various tumors, including colorectal cancer, hepatocellular carcinoma, and non-small-cell lung cancer, thereby promoting cell migration and invasion. Research indicates that HMGB1 activates the phosphatidylinositol 3-kinase (PI3K)/protein kinase B (PKB) pathway via BRG1, thereby facilitating EMT progression in lung epithelial cells. GLY intervenes in this process by impeding the interaction between HMGB1 and Brahma-related gene 1 (BRG1) through the PI3K/AKT/Mechanistic target of rapamycin (mTOR) pathway, thereby inhibiting EMT [93]. Additionally, GLY can directly bind to HMGB1, curtailing its expression and restraining the release of other pro-inflammatory cytokines such as TNF-α and IL-6, thus exerting anti-inflammatory effects.

GLY has a favorable inhibitory effect on bacteria, making it an important raw material for a variety of applications in healthcare and agriculture. The antimicrobial mechanism of GLY against pathogenic bacteria invading the human body has been characterized both in vivo and in vitro. In addition, its synergistic effect in combination with other drugs further enhances its efficacy against multidrug-resistant pathogens. This paper may provide a reference for the study of the in vitro and in vivo antimicrobial mechanisms of GLY as well as the combination of GLY with other drugs, and GLY has a promising future in increasing drug pathogenicity. The continued exploration of the properties and potential applications of GLY will hold great promise for improving public health and food safety.

## Figures and Tables

**Table 1:** Antibacterial mechanism of GLY in vitro.

Bacteria	Mechanism	References
*Klebsiella pneumoniae*	Inhibiting the activity of *K. pneumoniae* aromatic amine N-acetyltransferase	[39]
*Helicobacter pylori*	Inhibiting NAT activity in *H. pylori* in vivo	[40]
*Streptococcus mutans*	Inhibiting biofilm formation and glucose transferase activity	[47,48,49,50]
*Pseudomonas aeruginosa*	Inhibiting cell membrane formation and changes in cell membrane permeability, reducing bacterial viability and efflux pump activity	[51,52,57,58]
*Enterococcus*	Reducing drug resistance in *Enterococci*	[59]

**Table 2:** Antibacterial mechanism of GLY in vivo.

Bacteria	Mechanism	References
*Pseudomonas aeruginosa*	Inhibits HMGB1 to reduce the expression of proinflammatory factors and promote the production of antimicrobial peptides at the edge of burn tissue	[51,52,58,64,75]
*Salmonella enterica*	Induces the expression of iNOS and NOX-1 to promote the production of NO and H₂O₂ and activate macrophages to participate in immune regulation	[60,79,82]
Coagulase-negative *staphylococci*	Regulates inflammation by regulating histamine and other inflammatory mediators	[62,70]
*Helicobacter pylori*	Inhibits HMGB1 to restore lysosomal activity	[65]
*Staphylococcus aureus*	Reduces the production of inflammatory factors by inhibiting the P38 signaling pathway	[76]

## Data Availability

Not applicable.
